# Risk Factors for Therapeutic Failure and One-Year Mortality in Patients with Intramedullary Nail-Associated Infection after Trochanteric and Subtrochanteric Hip Fracture Repair

**DOI:** 10.3390/antibiotics13050463

**Published:** 2024-05-18

**Authors:** Bernadette Pfang, Marco A. Villegas García, Antonio Blanco García, Álvaro Auñón Rubio, Jaime Esteban, Joaquín García Cañete

**Affiliations:** 1 Unidad de Innovación Clínica y Organizativa, Red Quirónsalud 4H, 28040 Madrid, Spain; 2 Instituto de Investigación Sanitaria Fundación Jiménez Díaz, UAM, 28040 Madrid, Spain; 3 Industrial Engineering Politecnic, University of Castilla-La Mancha, 13071 Ciudad Real, Spain; 4 Emergency Department, Hospital Fundación Jiménez Díaz, 28040 Madrid, Spain; 5 CIBERINFEC-CIBER de Enfermedades Infecciosas, 28029 Madrid, Spain; 6 Orthopedic Surgery and Traumatology Department, Hospital Fundación Jiménez Díaz, 28040 Madrid, Spain

**Keywords:** hip osteosynthesis, infection, intramedullary nail, pertrochanteric fracture, subtrochanteric fracture, healthcare-associated infection

## Abstract

Despite the implications of trochanteric and subtrochanteric intramedullary (IM) nail infection for patients with hip fracture, little is known about risk factors for therapeutic failure and mortality in this population. We performed a retrospective observational analysis including patients diagnosed with trochanteric and subtrochanteric IM nail infection at a Spanish academic hospital during a 10-year period, with a minimum follow-up of 22 months. Of 4044 trochanteric and subtrochanteric IM nail implants, we identified 35 cases of infection during the study period (0.87%), 17 of which were chronic infections. Patients with therapeutic failure (n = 10) presented a higher average Charlson Comorbidity Index (CCI) (5.40 vs. 4.21, *p* 0.015, CI 0.26–2.13) and higher rates of polymicrobial (OR 5.70, *p* 0.033, CI 1.14–28.33) and multidrug-resistant (OR 7.00, *p* 0.027, CI 1.24–39.57) infections. Upon multivariate analysis, polymicrobial infection and the presence of multidrug-resistant pathogens were identified as independent risk factors for therapeutic failure. Implant retention was associated with an increased risk of failure in chronic infection and was found to be an independent risk factor for overall one-year mortality in the multivariate analysis. Our study highlights the importance of broad-spectrum empirical antibiotics as initial treatment of trochanteric and subtrochanteric IM nail-associated infection while awaiting microbiological results. It also provides initial evidence for the importance of implant removal in chronic IM-nail infection.

## 1. Introduction

The surgical treatment of trochanteric and subtrochanteric hip fractures is often performed using intramedullary nailing [1,2]. The potential complications of intramedullary nailing include mechanical complications such as cutout, early implant fracture, and delayed union [3,4] and medical complications such as bleeding, pulmonary embolism, and implant-associated infection [5,6]. Deep surgical site infection after intramedullary nailing of these fractures is uncommon, with an incidence of around 1–1.5% [7,8,9]. However, these infections lead to worse patient outcomes and increased healthcare expenditure due to additional surgical interventions, extended hospital stays, and long periods of antibiotic treatment [10,11]. 

To achieve infection control and bone union, most orthopedic implant-associated infections require surgical debridement with or without implant removal, as well as guided antibiotic therapy [12,13,14]. Due to the nature of trochanteric and subtrochanteric hip fractures, infected intramedullary (IM) nails must be retained or exchanged for other implants to preserve joint function and provide stability to allow the fracture to heal [15]. However, evidence for best practice in treatment of these infections is scarce, and due to the lack of consensus regarding optimal treatment [7,16], clinicians often base treatment strategies on guidelines for prosthetic joint infection of the hip [12]. The few reports present in the literature indicate that implant retention could be a valid option for IM nail-associated infection [7], although with worse outcomes than for other implants [12].

Our study describes a cohort of patients with trochanteric and subtrochanteric IM nail-associated infection from a tertiary level hospital in Spain, aiming to describe risk factors for therapeutic failure and one-year mortality in this population. 

## 2. Materials and Methods

We performed a retrospective observational study including patients diagnosed with trochanteric and subtrochanteric IM nail-associated infection at a tertiary hospital in Madrid, Spain during a 10-year period. 

All patients diagnosed with IM nail-associated infection after trochanteric or subtrochanteric hip fracture repair from 1 January 2011 to 31 December 2021, in the Fundación Jiménez Díaz University Hospital, a 686-bed tertiary hospital in Madrid, Spain, were included. Patients diagnosed with superficial surgical site infection and those who did not attend regular follow-up visits at the outpatient orthopedic surgery clinic were excluded from this study. The study design included the entire population, and so we did not perform a sample size calculation prior to data collection.

Data were collected manually from patients’ clinical records using the hospital’s electronic health record, Casiopea^®^ (Inetum, Saint-Ouen, France). Clinical records were reviewed until 1 November 2023, or until a patient’s death, with a minimum follow-up period of 22 months. Variables included demographic and clinical characteristics such as age, sex, and comorbidities; implant-related characteristics such as date of diagnosis, type of fracture, and type of intramedullary nail; and infection-related characteristics such as signs and symptoms of infection, blood test results, microbiological results, antibiotic therapy, surgical treatment (implant retention or removal), one-year mortality, attributable mortality, and infection control. Overall health status was calculated using the Charlson Comorbidity Index [17]. 

In line with the definition of fracture-related infection proposed by Metsemakers et al. [18], we defined an IM nail-associated infection as the presence of wound discharge, fistulae, or two phenotypically indistinguishable pathogens identified from surgically obtained tissue or hardware specimens. Suggestive signs of infection, such as fever, pain, and elevation of serum acute phase reactants, were collected. All patients received empirical antibiotic therapy with vancomycin (1 g/12 h) plus ceftazidime (2 g/8 h). In cases of previous antibiotic therapy, which can cause false-negative microbiological culture results, positive cultures were not required to demonstrate infection. Acute infections were considered as those diagnosed less than 30 days after hardware implantation without presence of a sinus tract [19]. Therapeutic failure was defined as a composite variable including persistent signs of infection (fistulae, persistent wound drainage, or elevated acute phase reactants), attributable death, or the decision to opt for suppressive antibiotic therapy.

Statistical analysis was performed in Python version 3.10, using the scipy.stats package from the SciPy v1.13.0 library and firthlogist 0.5.0. Continuous variables are expressed as mean (SD) and range or median (IQR) and range for normal and non-normal distributions, respectively. Categorical variables are presented as absolute values and percentages of the total sample. To compare differences between groups, we used Fisher’s exact test for categorical variables and Student’s t-test for continuous variables. To identify variables associated with therapeutic failure and one-year mortality, we performed multivariate analysis using Firth’s penalized linear regression [20] including those variables which had demonstrated statistical significance in the univariate analysis. Firth’s penalized linear regression was chosen because it is a more robust method for analyzing small datasets than traditional logistic regression, including those datasets including rare events and complete separation. A two-sided *p*-value of less than 0.05 was considered to indicate statistical significance. This study was approved by the institutional ethics committee (EO18/2014_FJD).

## 3. Results

During the study period (1 January 2011 to 31 December 2021), 4044 trochanteric hip fractures were treated with IM nails at the Fundación Jiménez Díaz University Hospital. We identified 35 cases of trochanteric IM nail infection during the study period. The overall incidence of implant-associated infection for IM nailing of trochanteric fractures was 0.87%.

Of the 35 patients diagnosed with IM nail-associated infection, one was excluded from analysis due to insufficient length of follow-up. The demographic and clinical characteristics of the remaining 34 patients are presented in Table 1. Of the 34 patients included in the analysis, 25 were female. Median age (IQR) was 87.79 (12.94) years, ranging from 39 to 99 years, with 24 patients aged 80 years or over at diagnosis. Intramedullary nails had been implanted in the context of fragility hip fractures in all but two patients (one pathological fracture due to bone metastasis and one pertrochanteric hip fracture due to a traffic accident). All implants were Gamma3^®^ nails (Stryker, MI, USA) except for one Proximal Femoral Nail Antirotation (PFNA^®^) (Synthes, West Chester, PE, USA). Thirty-one patients presented at least one comorbidity. The most frequent comorbid conditions included hypertension (19), atrial fibrillation (10), dyslipidemia (8), diabetes mellitus (7), dementia (7), heart failure (6), and liver failure (4). Patients scored an average of 5 (1.6, range 1–8) points on the Charlson Comorbidity Index (CCI), with 28 patients presenting a CCI of 5 or more points. Mean serum albumin levels at diagnosis were 2.9 g/dL (0.69, range 1.6–4.5 g/dL). 

The average time from intramedullary nail implantation to the diagnosis of infection was 178.85 days (401.24, range 7–2213 days). Seventeen infections were classified as chronic (>30 days from implantation). Signs and symptoms of infection included wound drainage (14), pain (10), fever (7), fistulae (7), erythema (5), fracture non-union (4), abscess (3), wound dehiscence (2), and bleeding (2). Regarding blood test results at diagnosis, patients presented an average white cell count of 8.29 × 10^9^/L (4.636, range 2.41–20.68 × 10^9^/L), neutrophil differential of 74.8% (12.3, 44.8–97.2%), and C-reactive protein levels of 9.72 mg/dL (10.52, 0.5 mg/dL–38.9 mg/dL).

Regarding microbiological characteristics of intramedullary nail-associated infection, 11 patients presented polymicrobial infections. Multidrug-resistant pathogens were detected in eight episodes. A total of 45 bacteria were isolated, of which 22 were gram-negative pathogens, including *Escherichia coli* (8, of which 2 were ESBL-producing strains), *Klebsiella pneumoniae* (5, of which 2 were ESBL-producing strains), *Enterobacter cloacae* (3), *Morganella morganii* (1), *Pseudomonas aeruginosa* (2), *Proteus mirabilis* (2), and *Providencia stuartii* (1) and 23 g-positive pathogens, including *Staphylococcus aureus* (11, 4 of them methicillin-resistant strains), *Enterococcus faecalis* (4), *Enterococcus faecium* (1), coagulase-negative *Staphylococci* (2), *Cutibacterium acnes* (2), Corynebacterium striatum (2), and *Listeria monocytogenes* (1). 

Combined antimicrobial treatment according to microbiological isolates was prescribed for 22 patients. The median (IQR) duration of antibiotic therapy was 56 days (24.5, range 4–360 days). Initially, the infected IM nail was removed in 11 patients, 1 patient underwent one-step (septic) implant exchange, 19 patients underwent surgical debridement, implant retention and antibiotics (DAIR), 1 patient was prescribed antibiotics with a curative intent, and 2 patients were directly prescribed chronic antibiotic suppression. Regarding definitive surgical strategy, IM nails were removed in 14 cases, while surgical debridement with implant retention was performed in 15, and chronic antibiotic suppression was prescribed in 5 cases. From the surgical point of view, DAIR failed in two cases (one acute and one chronic infection), as did the only case of septic one-step exchange. Of the 14 patients with definitive implant removal, 9 presented fracture healing at follow-up, while 2 did not present fracture healing, 1 patient died during hospital admission, and 2 patients underwent successful joint replacement after completing antibiotic treatment. One patient failing to present fracture healing came from the treatment failure group.

Therapeutic failure to control infection occurred in 10 cases. Demographic and clinical characteristics of patients with controlled and uncontrolled infection are presented in Table 2. Upon univariate analysis, the presence of multidrug-resistant pathogens (OR 7.00, *p* 0.027, CI 1.24–39.57) and polymicrobial infection (OR 5.70, *p* 0.033, CI 1.14–28.33) were found to be significantly associated with failure to control infection. Also, comorbidity was significantly higher in the group of patients with therapeutic failure, as demonstrated by a higher average CCI (5.40 vs. 4.21, *p* 0.015, CI 0.26–2.13). Both the presence of multidrug-resistant bacteria and polymicrobial infection were confirmed to be independent risk factors for therapeutic failure upon multivariate analysis. 

Regarding one-year mortality, eight patients died within 1 year of diagnosis of infection, with three patients presenting attributable mortality, all of whom died within 2 months of diagnosis. Variables associated with one-year mortality included implant retention (OR 19.72, *p* 0.0478, CI 1.03–377.08) and uncontrolled IM nail infection (OR 7.00, *p* 0.027, CI 1.24–39.57). Mean CCI was significantly higher in the group of patients who died within the first year after diagnosis of infection (5.62 vs. 4.23, *p* 0.006, CI 0.45–2.33). However, upon multivariate analysis, only implant retention was found to be an independent risk factor for one-year mortality. 

We conducted a subgroup analysis of patients with chronic IM nail infection, which demonstrated a significant association between implant retention and failure to achieve infection control (OR 20, *p* 0.028, CI 1.39–287.61). On the other hand, for the group of patients with acute IM nail infection, implant retention was not associated with therapeutic failure. 

## 4. Discussion

Our study describes risk factors for therapeutic failure and one-year mortality in a cohort of patients diagnosed with trochanteric and subtrochanteric IM nail infection at a major Spanish academic hospital during a ten-year period. During the study period, the overall incidence of trochanteric and subtrochanteric IM nail infection was 0.87%, slightly lower than in other series [7,8,9]. Patients were mostly aged over 75 years and presented high levels of comorbidity, consistent with other studies [2,21,22,23]. Therapeutic failure occurred in 10 cases of infection (29.41%), and 8 patients (23.53%) died within one year from diagnosis. Upon multivariate analysis, independent risk factors for therapeutic failure included presence of multidrug-resistant pathogens and polymicrobial infection, while independent risk factors associated with one-year mortality included implant retention and uncontrolled infection. In patients with chronic infection, implant retention was associated with a higher risk of therapeutic failure. 

To the best of our knowledge, this is the largest cohort to feature patients with trochanteric and subtrochanteric IM nail-associated infection. Existing studies reporting prevalence and risk factors for infection after IM nailing of trochanteric fractures include patients with both superficial and deep surgical site infection [7,9], whereas our study focuses exclusively on deep surgical site infection. Despite its relatively small sample size, the homogenous nature of our cohort allowed us to perform an analysis of risk factors for therapeutic failure and one-year mortality, which has not been reported previously in the literature. 

This study has several limitations. The retrospective study design leads to higher risk of bias than prospective studies due to various factors, including selection bias. To mitigate the risk of selection bias, we included all cases of trochanteric and subtrochanteric IM nail-associated infection, which were registered prospectively by the hospital’s Bone and Joint Infection Team. Incomplete or insufficient follow-up can also bias the results of retrospective studies like this one, and so we only included patients who completed a minimum of 24 months of follow-up with regular appointments at our center. To minimize risk of heterogeneity regarding data entry, data extraction and entry was performed by one researcher and checked by other investigators to ensure accuracy. Apart from its retrospective design, the main limitation of our research is the length of the inclusion period, which could potentially over- or under-estimate the relevance of certain variables such as the presence of multidrug-resistant pathogens due to time-related changes in prevalence. However, due to the low prevalence of orthopedic implant-associated infection, ten-year inclusion periods are common in this field of research [24,25,26,27]. Moreover, as the therapeutic approach to trochanteric IM nail-associated infection has not changed significantly in our center over the last decade, we believe that the probability of bias due to the length of the study is minimal. Finally, in our center, the standard implant for pertrochanteric hip fracture is the Gamma3 nail (Stryker, MI, USA). Consequently, our cohort featured mainly gamma nails, and we were unable to compare outcomes for different implants.

An incorrect choice of empirical antibiotic treatment has been described as a risk factor for treatment failure in orthopedic implant-associated infection [28]. Our center’s protocol for empirical treatment is similar to that of other Spanish hospitals [29] and comprises vancomycin 1 g c/12 h and ceftazidime 2 g c/8 h until microbiological results are available. The high prevalence of polymicrobial infection (32.5%) in our study, as well as the frequent detection of gram-negative and multidrug-resistant pathogens, point to the importance of broad-spectrum empiric antibiotics covering both gram-positive and gram-negative pathogens while awaiting results from microbiological cultures to guide directed therapy, as has been demonstrated in several studies [30,31]. Studies on prosthetic joint infection have demonstrated an increase in both gram-negative and multidrug-resistant pathogens in recent years [32,33]. Fifty percent of isolates in this study were gram-negative bacteria, while multidrug-resistant bacteria were isolated in almost a quarter of infections, demonstrating higher prevalences for these pathogens than those reported for prosthetic joint infections (10–33.3% and 12.5–15.8%, respectively [32,33,34,35,36]). However, when interpreting these data, it is important to consider the older age and high comorbidity of patients included in the study, as both age and comorbidity have been described as risk factors for both gram-negative and multidrug-resistant infections [37,38]. 

According to our results, therapeutic failure is common in patients with trochanteric and subtrochanteric IM nail-associated infection. Independent risk factors for therapeutic failure included the presence of polymicrobial infection and multidrug-resistant pathogens, findings which mirror those observed for prosthetic joint infection [39,40]. Higher average CCI scores were found in the group of patients who failed to achieve infection control. This finding is consistent with other studies which report an association between comorbidities and poorer outcomes for surgical site infection [41,42]. However, upon multivariate analysis, higher CCI scores were not found to be independent risk factors for therapeutic failure, perhaps due to the fact that burden of comorbidity has been found to correlate to prevalence of polymicrobial infection and multidrug-resistant pathogens [43,44]. However, it is also possible that the small sample size was the reason for CCI not proving significant in the multivariate analysis. 

Although implant retention was not related to worse outcomes in the global analysis, for those patients with chronic implant-associated infection, IM nail retention was significantly associated with a higher risk for failure. Although sample size is an evident limitation of these findings, our results are in line with research on prosthetic joint infection which demonstrates that the duration of infection is associated with lower chances of success when attempting a debridement, antibiotics, and implant retention strategy (DAIR) [45], and point to the importance of implant removal in patients with chronic infection, as the formation of biofilms can impede eradication of bacteria despite antibiotics and debridement [46,47]. A recent single-center study from Finland reporting superficial and deep infections after intramedullary fixation of trochanteric and subtrochanteric fractures, observed that none of the deep infections included (n = 15) required implant removal [7]. However, in this series, only three infections presented after four weeks from initial surgery, pointing to a much lower prevalence of chronic infections than in our sample. 

Early surgical site infection has been associated with increased mortality after hip fracture repair [23,48]. A previous, single-center retrospective study set in Austria found an association between gram-positive microorganisms such as Staphylococcus aureus (including methicillin-resistant strains) and Enterococcus spp. and higher rates of mortality in patients with infection after trochanteric or subtrochanteric fractures treated with osteosynthesis [9]. However, this association was not observed in our series. The relatively high rate of one-year mortality after diagnosis of infection observed in our cohort indicates that IM nail infection may be associated with higher mortality regardless of time since the original surgery. Implant retention and therapeutic failure were both found to be independent risk factors for mortality one year after diagnosis. Opting to remove a trochanteric or subtrochanteric IM nail is often a difficult decision, in which multiple factors—the patient’s overall physical status, clinical condition, and preferences, and the surgeon’s expertise—must be taken into account [49]. However, two variables which are often considered when choosing to retain an implant (age and comorbidity) were not found to be associated with one-year mortality in our analysis. Although our results must be interpreted with caution due to the small sample size, they point to a potential association between the retention of an infected IM nail and one-year mortality that merits further research. 

The incidence of trochanteric and subtrochanteric hip fracture is predicted to increase over the coming decades due to an aging population and higher prevalence of risk factors for hip fracture. Thus, although rates of surgical site infection present a decreasing trend, the incidence of IM nail infections will potentially increase over time, underlining the importance of developing strategies for prevention, timely diagnosis, and effective treatment. Despite the single-center setting of our study and its limitations including its retrospective design our study provides further evidence supporting broad spectrum antibiotics targeting both gram-positive and gram-negative infections until cultures are available to direct the choice of antibiotic treatment. Patients with comorbidities present higher rates of therapeutic failure, and careful evaluation and treatment of comorbidities could potentially improve chances of infection control. Our findings also suggest that implant retention should be avoided in chronic infection, although further research is needed to confirm these findings in the general population.

## 5. Conclusions

Trochanteric and subtrochanteric IM nail-associated infection is a rare but devastating complication of hip fracture repair, and treatment is often complex. Risk factors for therapeutic failure include polymicrobial infection and multidrug-resistant pathogens, pointing to the importance of broad-spectrum empirical antibiotics as initial treatment while awaiting the results of microbiological cultures. Implant retention is associated with an increased risk of failure in chronic infection, as well as with overall higher one-year mortality. Further studies are necessary to confirm these results.

## Figures and Tables

**Table 1:** Demographic and clinical characteristics of patients diagnosed with trochanteric IM nail-associated infection during the study period.

Patient	Sex	Age	Comorbidities	CCI	Acute/Chronic	Days from Implant to Infection Diagnosis	Pathogen	Initial Surgical Treatment	Definitive Surgical Treatment	Antibiotic/Duration (Days)	Combined Antibiotic Therapy	One-Year Mortality/Attributable Mortality	Infection Control	Fracture Healing
1	M	39	Ulcerative colitis	1	C	196	*Enterococcus faecium*	Implant removal	Implant removal	Amoxicillin-clavulanic acid/90	No	No/No	Yes	Yes
2	F	59	Obesity, sleep apnea, lymphoma	3	C	310	*Corynebacterium striatum*; *Escherichia coli*	Implant removal	Implant removal	Fosfomycin and co-trimoxazole/90	Yes	No/No	Yes	N/A (total hip replacement was carried out after full course of antibiotic treatment)
3	M	67	Dyslipidemia	2	C	442	MSSA	Implant removal	Implant removal	Ciprofloxacin and co-trimoxazole/35	Yes	No/No	Yes	Yes
4	F	68	Hypertension, dysplidemia, hypothyroidism, osteoporosis	2	A	24	MSSA	DAIR	DAIR	Levofloxacin and rifampicin/360	Yes	No/No	Yes	N/A
5	M	68	Hypertension, atrial fibrillation, liver failure	5	A	9	*Enterobacter cloacae*	DAIR	DAIR	Imipenem and ciprofloxacin/30	Yes	Yes/Yes	Yes	N/A
6	M	78	Hypertension, T2DM, mielodysplasic syndrome	4	A	30	*Pseudomonas aeruginosa*	DAIR	DAIR	Ciprofloxacin and imipenem/42	Yes	No/No	Yes	N/A
7	F	79	Atrial fibrillation, heart failure, liver failure	7	C	53	ESBL-producing *Klebsiella pneumoniae*; *Providencia stuartii*	Suppressive antibiotic therapy	Suppressive antibiotic therapy	Ciprofloxacin/suppressive	No	Yes/No	No	N/A
8	F	82	Dyslipidemia	4	A	7	Coagulase-negative *Staphylococcus*	DAIR	Implant removal	Levofloxacin and rifampicin/84	Yes	No/No	Yes	N/A
9	M	82	Hypertension, atrial fibrillation, mild cognitive impairment	5	A	23	MSSA	DAIR	DAIR	Ciprofloxacin and rifampicin/42	Yes	No/No	Yes	Yes
10	F	86	Hypertension, moderate cognitive impairment	5	C	49	*Pseudomonas aeruginosa*; *Corynebacterium striatum*; *MSSA*	DAIR	Implant removal	Linezolid and rifampicin/90	Yes	No/No	No	No
11	F	86	Hypertension	4	C	223	*Listeria monocytogenes*	Implant removal	Implant removal	Co-trimoxazole/42	No	No/No	Yes	Yes
12	F	88	Hypertension, dyslipidemia, atrial fibrillation, heart failure	5	A	21	*Enterobacter cloacae*	DAIR	DAIR	Imipenem and fosfomycin/42	Yes	No/No	Yes	N/A
13	F	88	Hypertension, dyslipidemia, atrial fibrillation, coronary artery disease, mild cognitive impairment	6	C	80	*Escherichia coli*; *Proteus mirabilis*	DAIR	DAIR	Levofloxacin and co-trimoxazole/70	Yes	Yes/Yes	No	N/A
14	F	89	Hypertension, heart failure	5	C	34	MRSA	Antibiotics with curative intent	Suppressive antibiotic therapy	Rifampicin and clindamycin/suppressive	Yes	No/No	No	N/A
15	F	89	Atrial fibrillation, heart failure, ischemic stroke, cognitive impairment	6	C	45	MSSA; coagulase-negative *Staphylococcus*	DAIR	DAIR	Fusidic acid and rifampicin/42	Yes	No/No	Yes	N/A
16	F	90	Atrial fibrillation, heart failure, cognitive impairment	6	A	10	*Klebsiella pneumoniae*	DAIR	Implant removal	Co-trimoxazole and ciprofloxacin/56	Yes	No/No	Yes	Yes
17	F	91	Hypertension, giant cell arteritis, ischemic stroke	6	A	19	MSSA; *Escherichia coli*	Implant removal	Implant removal	Cefazolin and gentamycin/56	Yes	Yes/No	No	N/A (early death during hospital admission)
18	F	91	T2DM, dyslipidemia	5	A	11	*Morganella morganii*; *Klebsiella pneumoniae*; *Enterobacter cloacae*	DAIR	DAIR	Levofloxacin/42	No	Yes/No	Yes	N/A
19	F	91		4	C	61	ESBL-producing *Escherichia coli*; *Enterococcus faecalis*	Implant removal	Implant removal	Fosfomycin, amoxicillin, and co-trimoxazole/90	Yes	No/No	Yes	N/A (partial hip replacement was performed a full course of antibiotic therapy)
20	F	91	Hypertension, chronic kidney disease	6	C	744	*Cutibacterium acnes*	Implant removal	Implant removal	Levofloxacin/56	No	No/No	Yes	Yes
21	F	96	Hypertension, dyslipidemia, T2DM, atrial fibrillation, heart failure	6	C	34	MRSA	Implant removal	Implant removal	Clindamycin/56	No	No/No	Yes	No
22	F	99	Venous insufficiency	4	C	51	Gut microbiota	Suppressive antibiotic therapy	Suppressive antibiotic therapy	Co-trimoxazole/suppressive	No	No/No	No	N/A
23	M	49	Alcohol abuse, liver failure, HIV infection, HCV infection	3	A	18	*Escherichia coli*	DAIR	DAIR	Amoxicillin-clavulanic acid and ciprofloxacin/42	Yes	No/No	Yes	N/A
24	M	68	T2DM, liver failure, HIV infection, HBV infection	6	A	23	ESBL-producing *Escherichia coli*	DAIR	DAIR	Imipenem/28	No	Yes/No	No	N/A
25	F	89	Hypertension	4	C	587	MSSA	One-step septic exchange	Implant removal	Levofloxacin and rifampicin/56	Yes	No/No	Yes	Yes
26	F	93	Hypertension	4	A	20	*Escherichia coli*	DAIR	Suppressive antibiotic therapy	Ciprofloxacin/suppressive	No	No/No	No	N/A
27	F	92	Hypertension, mild cognitive impairment	5	C	138	ESBL-producing *Klebsiella pneumoniae*; *Proteus mirabilis*	Implant removal	Suppressive antibiotic therapy	Ertapenem and ciprofloxacin/52; then switched to ciprofloxacin/suppressive	Yes	No/No	No	N/A
28	M	93	Hypertension, atrial fibrillation	4	A	27	MRSA	DAIR	DAIR	Clindamycin and rifampicin/90	Yes	No/No	Yes	N/A
29	M	55		1	C	2213	*Cutibacterium acnes*	Implant removal	Implant removal	Clindamycin and rifampicin/60	Yes	No/No	Yes	Yes
30	F	84	Hypertension, atrial fibrillation	4	C	343	*Enterococcus faecalis*	Implant removal	Implant removal	Amoxicillin/35	No	No/No	Yes	Yes
31	F	88	Hypertension, T2DM, dyslipidemia	5	A	11	*Enterococcus faecalis*	DAIR	DAIR	Amoxicillin/56	No	No/No	Yes	N/A
32	F	96	T2DM, atrial fibrillation, cognitive impairment	6	A	13	MRSA	DAIR	DAIR	Vancomycin and clindamycin/4	Yes	Yes/Yes	No	N/A
33	F	93	T2DM, coronary artery disease, heart failure	8	A	15	*Escherichia coli*; *Enterococcus faecalis*; *Klebsiella pneumoniae*	DAIR	DAIR	Amoxicillin/60	No	No/No	Yes	N/A
34	F	93		4	A	27	Culture negative (prior antibiotic therapy)	DAIR	DAIR	Ciprofloxacin and clindamycin/60	Yes	Yes/No	Yes	N/A

T2DM, Type 2 diabetes mellitus; HIV, human immunodeficiency virus; HBV, hepatitis B virus; HCV, hepatitis C virus; ESBL, extended spectrum betalactamase; MRSA, methicillin-resistant *Staphylococcus* aureus; MSSA, methicillin-susceptible *Staphylococcus aureus*; DAIR, debridement, antibiotics, and implant retention.

**Table 2:** Demographic and clinical characteristics of patients with intramedullary nail infection of the hip, comparing patients with controlled and uncontrolled infection.

	Infection Control (n = 24)	Therapeutic Failure (n = 10)
Female	16 (66.7%)	9 (90.0%)
Age	79.6 (SD 15.7)	88.1 (SD 9.03)
Comorbidities	21 (87.5%)	10 (100.0%)
Charlson Comorbidity Index *	4.2 (SD 2.8)	5.4 (SD 0.93)
Chronic infection	13 (54.2%)	4 (40.0%)
Polymicrobial infection *	5 (20.8%)	6 (60.0%)
Multidrug-resistant pathogen *	3 (12.5%)	5 (50.0%)
Implant removal	12 (50.0%)	2 (20.0%)
Combined antibiotic treatment	16 (66.7%)	6 (60.0%)

* *p* < 0.05.

## Data Availability

Data are available upon reasonable request.

## References

[1] Sáez-López P., Ojeda-Thies C., Alarcón T., Muñoz Pascual A., Mora-Fernández J., González de Villaumbrosia C., Molina Hernández M.J. (2019). Spanish National Hip Fracture Registry (RNFC): First-year Results and Comparison with Other Registries and Prospective Multicentric Studies from Spain. Rev. Esp. Salud Pública.

[2] Schemitsch E.H., Nowak L.L., Schulz A.P., Brink O., Poolman R.W., Mehta S., Stengel D., Zhang C.Q., Martinez S., Kinner B. (2023). Intramedullary Nailing vs. Sliding Hip Screw in Trochanteric Fracture Management: The INSITE Randomized Clinical Trial. JAMA Netw. Open.

[3] Yoon J.Y., Park S., Kim T., Im G.I. (2022). Cut-out risk factor analysis after intramedullary nailing for the treatment of extracapsular fractures of the proximal femur: A retrospective study. BMC Musculoskelet. Disord..

[4] Klima M.L. (2022). Mechanical Complications After Intramedullary Fixation of Extracapsular Hip Fractures. J. Am. Acad. Orthop. Surg..

[5] Lähdesmäki M., Ylitalo A.A., Karjalainen L., Uimonen M., Mattila V.M., Repo J.P. (2023). Intramedullary Nailing of Intertrochanteric Femoral Fractures in a Level I Trauma Center in Finland: What Complications Can be Expected?. Clin. Orthop. Relat. Res..

[6] Panteli M., Vun J.S., Ahmadi M., West R.M., Howard A.J., Chloros G., Pountos I., Giannoudis P.V. (2023). Blood loss and transfusion risk in intramedullary nailing for subtrochanteric fractures. Transfus. Med..

[7] Halonen L.M., Stenroos A., Vasara H., Huotari K., Kosola J. (2021). Infections after intramedullary fixation of trochanteric fractures are uncommon and implant removal is not usually needed. Injury.

[8] Evaniew N., Bhandari M. (2015). Cochrane in CORR^®^: Intramedullary Nails for Extracapsular Hip Fractures in Adults (Review). Clin. Orthop. Relat. Res..

[9] Sator T., Binder H., Payr S., Pichler L., Frenzel S., Hajdu S., Presterl E., Tiefenboeck T.M. (2024). Surgical site infection after trochanteric and subtrochanteric fractures: A single centre retrospective analysis. Sci. Rep..

[10] Zimlichman E., Henderson D., Tamir O., Franz C., Song P., Yamin C.K., Keohane C., Denham C.R., Bates D.W. (2013). Health Care–Associated Infections: A Meta-analysis of Costs and Financial Impact on the US Health Care System. JAMA Intern. Med..

[11] Whitehouse J.D., Friedman N.D., Kirkland K.B., Richardson W.J., Sexton D.J. (2002). The impact of surgical-site infections following orthopedic surgery at a community hospital and a university hospital: Adverse quality of life, excess length of stay, and extra cost. Infect. Control Hosp. Epidemiol..

[12] Metsemakers W.J., Kuehl R., Moriarty T.F., Richards R.G., Verhofstad M.H.J., Borens O., Kates S., Morgenstern M. (2018). Infection after fracture fixation: Current surgical and microbiological concepts. Injury.

[13] Hellebrekers P., Leenen LP H., Hoekstra M., Hietbrink F. (2017). Effect of a standardized treatment regime for infection after osteosynthesis. J. Orthop. Surg. Res..

[14] Hellebrekers P., Verhofstad M.H., Leenen L.P., Varol H., van Lieshout E.M., Hietbrink F. (2020). The effect of early broad-spectrum versus delayed narrow-spectrum antibiotic therapy on the primary cure rate of acute infection after osteosynthesis. Eur. J. Trauma Emerg. Surg..

[15] Zimmerli W. (2014). Clinical presentation and treatment of orthopaedic implant-associated infection. J. Intern. Med..

[16] Simpson A.H., Tsang J.S.T. (2017). Current treatment of infected non-union after intramedullary nailing. Injury.

[17] D’Hoore W., Sicotte C., Tilquin C. (1993). Risk adjustment in outcome assessment: The Charlson comorbidity index. Methods Inf. Med..

[18] Metsemakers W.J., Morgenstern M., McNally M.A., Moriarty T.F., McFadyen I., Scarborough M., Athanasou N.A., Ochsner P.E., Kuehl R., Raschke M. (2018). Fracture-related infection: A consensus on definition from an international expert group. Injury.

[19] Sukhonthamarn K., Tan T.L., Xu C., Kuo F.C., Lee M.S., Citak M., Gehrke T., Goswami K., Parvizi J. (2020). Determining Diagnostic Thresholds for Acute Postoperative Periprosthetic Joint Infection. J. Bone Jt. Surg..

[20] Firth D. (1993). Bias reduction of maximum likelihood estimates. Biometrika.

[21] Grønhaug K.M.L., Dybvik E., Matre K., Östman B., Gjertsen J.E. (2023). Comparison of Intramedullary Nails in the Treatment of Trochanteric and Subtrochanteric Fractures: An Observational Study of 13,232 Fractures in the Norwegian Hip Fracture Register. J. Bone Jt. Surg..

[22] Horner N.S., Samuelsson K., Solyom J., Bjørgul K., Ayeni O.R., Östman B. (2017). Implant-Related Complications and Mortality After Use of Short or Long Gamma Nail for Intertrochanteric and Subtrochanteric Fractures: A Prospective Study with Minimum 13-Year Follow-up. JBJS Open Access.

[23] Velez M., Palacios-Barahona U., Paredes-Laverde M., Ramos-Castaneda J.A. (2020). Factors associated with mortality due to trochanteric fracture. A cross-sectional study. Orthop. Traumatol. Surg. Res..

[24] Fischbacher A., Borens O. (2019). Prosthetic-joint infections: Mortality over the last 10 years. J. Bone Jt. Infect..

[25] Lenguerrand E., Whitehouse M.R., Beswick A.D., Kunutsor S.K., Burston B., Porter M., Blom A.W. (2018). Risk factors associated with revision for prosthetic joint infection after hip replacement: A prospective observational cohort study. Lancet Infect. Dis..

[26] Henry T.W., McEntee R.M., Matzon J.L., Beredjiklian P.K., Lutsky K.F. (2021). Deep Infection after Distal Radius Open-reduction Internal Fixation: A Case Series. Arch. Bone Jt. Surg..

[27] Chalmers B.P., Weston J.T., Hanssen A.D., Berry D.J., Abdel M.P., Osmon D.R. (2019). Prior hip or knee prosthetic joint infection in another joint increases risk three-fold of prosthetic joint infection after primary total knee arthroplasty: A matched control study. Bone Jt. J..

[28] Soriano A., Marco F., Martínez J.A., Pisos E., Almela M., Dimova V.P., Alamo D., Ortega M., Lopez J., Mensa J. (2008). Influence of vancomycin minimum inhibitory concentration on the treatment of methicillin-resistant Staphylococcus aureus bacteremia. Clin. Infect. Dis. Off. Publ. Infect. Dis. Soc. Am..

[29] Tornero E., Morata L., Martínez-Pastor J.C., Bori G., Climent C., García-Velez D.M., García-Ramiro S., Bosch J., Mensa J., Soriano A. (2015). KLIC-score for predicting early failure in prosthetic joint infections treated with debridement, implant retention and antibiotics. Clin. Microbiol. Infect. Off. Publ. Eur. Soc. Clin. Microbiol. Infect. Dis..

[30] Scholten R., Klein Klouwenberg P.M.C., Gisolf J.E.H., van Susante J.L.C., Somford M.P. (2023). Empiric antibiotic therapy in early periprosthetic joint infection: A retrospective cohort study. Eur. J. Orthop. Surg. Traumatol..

[31] Moran E., Masters S., Berendt A.R., McLardy-Smith P., Byren I., Atkins B.L. (2007). Guiding empirical antibiotic therapy in orthopaedics: The microbiology of prosthetic joint infection managed by debridement, irrigation and prosthesis retention. J. Infect..

[32] Benito N., Franco M., Ribera A., Soriano A., Rodriguez-Pardo D., Sorlí L., Fresco G., Fernández-Sampedro M., Del Toro M.D., Guío L. (2016). Time trends in the aetiology of prosthetic joint infections: A multicentre cohort study. Clin. Microbiol. Infect..

[33] Siljander M.P., Sobh A.H., Baker K.C., Baker E.A., Kaplan L.M. (2018). Multidrug-Resistant Organisms in the Setting of Periprosthetic Joint Infection—Diagnosis, Prevention, and Treatment. J. Arthroplast..

[34] Zmistowski B., Fedorka C.J., Sheehan E., Deirmengian G., Austin M.S., Parvizi J. (2011). Prosthetic Joint Infection Caused by Gram-Negative Organisms. J. Arthroplast..

[35] Casenaz A., Piroth L., Labattut L., Sixt T., Magallon A., Guilloteau A., Neuwirth C., Amoureux L. (2022). Epidemiology and antibiotic resistance of prosthetic joint infections according to time of occurrence, a 10-year study. J. Infect..

[36] Benito N., Mur I., Ribera A., Soriano A., Rodriguez-Pardo D., Sorli L., Cobo J., Fernandez-Sampedro M., Del Toro M.D., Guío L. (2019). The Different Microbial Etiology of Prosthetic Joint Infections according to Route of Acquisition and Time after Prosthesis Implantation, Including the Role of Multidrug-Resistant Organisms. J. Clin. Med..

[37] Pfang B.G., García-Cañete J., García-Lasheras J., Blanco A., Auñón Á., Parron-Cambero R., Macías-Valcayo A., Esteban J. (2019). Orthopedic Implant-Associated Infection by Multidrug Resistant Enterobacteriaceae. J. Clin. Med..

[38] Hsieh P.H., Lee M.S., Hsu K.Y., Chang Y.H., Shin H.N., Ueng S.W. (2009). Gram-negative prosthetic joint infections: Risk factors and outcome of treatment. Clin. Infect. Dis..

[39] Sadique H., Evans S., Parry M., Stevenson J., Reeves N., Mimmack S., Jumaa P., Jeys L. (2016). Multidrug-resistant bacteria: An independent predictor of failure in peri-prosthetic joint infection. Orthop. Proc..

[40] Rudelli B.A., Giglio P.N., de Carvalho V.C., Pécora J.R., Gurgel H.M.C., Gobbi R.G., Vicente J.R.N., Lima A.L.L.M., Helito C.P. (2020). Bacteria drug resistance profile affects knee and hip periprosthetic joint infection outcome with debridement, antibiotics and implant retention. BMC Musculoskelet. Disord..

[41] Koch K.A., Spranz D.M., Westhauser F., Bruckner T., Lehner B., Alvand A., Merle C., Walker T. (2023). Impact of Comorbidities and Previous Surgery on Mid-Term Results of Revision Total Knee Arthroplasty for Periprosthetic Joint Infection. J. Clin. Med..

[42] Payá-Llorente C., Martínez-López E., Sebastián-Tomás J.C., Santarrufina-Martínez S., de’Angelis N., Martínez-Pérez A. (2020). The impact of age and comorbidity on the postoperative outcomes after emergency surgical management of complicated intra-abdominal infections. Sci. Rep..

[43] Picot-Guéraud R., Batailler P., Caspar Y., Hennebique A., Mallaret M.R. (2015). Bacteremia caused by multidrug-resistant bacteria in a French university hospital center: 3 years of collection. Am. J. Infect. Control.

[44] Laudisio A., Marinosci F., Fontana D., Gemma A., Zizzo A., Coppola A., Rodano L., Antonelli Incalzi R. (2015). The burden of comorbidity is associated with symptomatic polymicrobial urinary tract infection among institutionalized elderly. Aging Clin. Exp. Res..

[45] Zhu M.F., Kim K., Cavadino A., Coleman B., Munro J.T., Young S.W. (2021). Success Rates of Debridement, Antibiotics, and Implant Retention in 230 Infected Total Knee Arthroplasties: Implications for Classification of Periprosthetic Joint Infection. J. Arthroplast..

[46] Zimmerli W., Sendi P. (2017). Orthopaedic biofilm infections. APMIS.

[47] Peel T.N. (2019). Studying biofilm and clinical issues in orthopedics. Front. Microbiol..

[48] Pollmann C.T., Dahl F.A., Røtterud JH M., Gjertsen J.E., Årøen A. (2020). Surgical site infection after hip fracture-mortality and risk factors: An observational cohort study of 1709 patients. Acta Orthop..

[49] Sousa R., Abreu M.A. (2018). Treatment of Prosthetic Joint Infection with Debridement, Antibiotics and Irrigation with Implant Retention—A Narrative Review. J. Bone Jt. Infect..

